# Atrophy of the Optic Nerve in Diseases of the Nervous System1Read before the Central New York Medical Association, Buffalo, N. Y., October 16, 1894.

**Published:** 1895-04

**Authors:** Alvin A. Hubbell

**Affiliations:** Professor of Ophthalmology and Otology in the Medical Department, Niagara University, Buffalo, N. Y.


### ATROPHY OF THE OPTIC NERVE IN DISEASES OF THE NERVOUS SYSTEM.[1]

BY ALVIN A. HUBBELL, M. D.,
Professor of Ophthalmology and Otology in the Medical Department, Niagara University, Buffalo, N. Y.

AFTER a careful study of optic neuritis, in regard to its etiology and its relations to other diseases, I came to the conclusion a few years ago that it was but one form of peripheral neuritis, and but an expression in the optic nerve, as in other peripheral nerves, of conditions and diseases of which this is but one symptom.

At this time I desire to call attention to another state of the optic nerve, which is also but a symptom and but one expression of manifold changes going on elsewhere in the central nervous system. I refer to primary atrophy, or gray atrophy, of the optic nerve.

There are two principal forms of optic-nerve atrophy. In one there has been a preceding inflammation of the nerve, more or less pronounced, and the remains of inflammatory products are more or less visible by the ophthalmoscope. The optic disc is at first grayish-white and its margins are slightly hazy, and the veins distended and tortuous. Afterwards its surface becomes a uniform pure white or bluish-white. It is sharply defined and often appears irregular in outline and smaller than normal, and both the arteries and veins are contracted.

In the other form there are no evidences of inflammatory action having taken place. The optic disc simply loses its normal "flush" and gradually becomes paler, till at length it is perfectly white or bluish-white, and its edges are sharply defined and slightly excavated. The gray dots of the lamina cribrosa become more visible and are seen over a larger area, appearing like "stippling." The minute vessels of the disc disappear, but the retinal arteries and veins are not markedly changed as in inflammatory atrophy.

It is this latter form of atrophy, not preceded by intraocular disease, not the sequence of inflammation of the optic nerve, not due to injury of, or pressure upon the optic nerve, and not caused by some specific poison in the blood, as lead, quinine, etc., to which I desire to direct attention now. It is known by the terms primary

---

1. Read before the Central New York Medical Association, Buffalo, N. Y., October 16, 1894.

atrophy, simple atrophy, gray atrophy. It is, too often, first recognized by the ophthalmologist, alone, through a consultation for impaired vision. This impairment of vision is shown in diminution of color-sense, contraction of the fields of vision or irregular defects in the fields, and in reduction of the acuity of vision. The loss of vision, however, is often in excess of the degree of atrophy, or rather, the vision fails more rapidly in some cases than the atrophy progresses.

In typical cases the ophthalmoscope shows the appearances of the optic discs above described. But this picture is not typical of all cases, and in the exceptional ones the color of the disc varies from mere paleness, limited, perhaps, to the temporal side, to an even whiteness without "stippling," or to the distinctively stippled bluish-white. This form of atrophy is diagnosed with the ophthalmoscope by the absence of inflammatory remains on the disc or at its edges, and by the more natural size of the retinal arteries and veins and their lack of tortuosity.

This simple, or gray atrophy, although a pathological state of the nerve, is in the vast majority, and I might, I think, say in all cases, but a symptom. As Dr. Buzzard, of London, truly says: "Atrophy of the optic nerve is evidently a symptom, not a disease; it is the visible result of a structural change which itself depends upon something more properly entitled to be considered the disease from which the patient is suffering" (*British Medical Journal*, October 7, 1893). It is as such a symptom of some other diseased condition that I wish to bring an emphasis upon it at this time, and a symptom worthy of more attentive watching on the part, especially, of the neurologist. Again, as a symptom, it is often one of the earliest to make its appearance in the course of certain grave diseases of the nervous system, and the impairment of vision usually attending it leads the patient to consult the ophthalmologist, who should at once recognize it, not as a disease *per se*, but merely as a symptom, like "paralysis," of some central nervous disease, and remand the patient to the neurologist or physician for treatment.

Those diseases in which optic atrophy so frequently becomes a symptom are, according to Uhthoff, who published the results of his carefully conducted studies in 1884, and of Charcot, Buzzard, Gowers and others:

1. Disseminated or insular sclerosis of the spinal cord and brain.

2. Tabes dorsalis or sclerosis of the posterior columns of the spinal cord.

3. General paralysis of the insane, with degeneration of the spinal cord.

4. Spastic paraplegia, or lateral sclerosis of the spinal cord.

The relation of atrophic changes to disseminated sclerosis has been more diligently studied by Dr. Uhthoff and Dr. Thos. Buzzard than by any other persons. They find this symptom more frequently in this disease than in any other, and until further investigations it is proper to accept their results. Uhthoff declares that with the exception of cerebral tumors and tuberculous meningitis there is no disease of the nervous system, not even tabes, which is so often accompanied by ophthalmoscopic changes as disseminated sclerosis. In some there is complete atrophy and loss of color of the disc ; in others incomplete discoloration, the nasal side preserving a slightly reddish reflection ; in others decoloration of the temporal side alone ; and in others still hyperemia with the appearances of inflammation. In 52 out of 100 cases the discs were abnormal. Buzzard found that 43 out of 100 cases of this disease had atrophic changes in the optic discs.

In tabes dorsalis, or locomotor ataxy, the percentage of cases of atrophy, according to Gowers and Buzzard, does not, probably, exceed fifteen. Nettleship found in seventy-six cases of optic atrophy thirty-eight cases presenting undoubted symptoms of locomotor ataxy, or 50 per cent. Gowers' experience included cases of locomotor ataxy, while Nettleship's referred to cases of optic atrophy. Putting the two together it shows the prominence which this symptom has in this disease. Atrophy of the optic disc appears in most cases of tabes in the early stage before the gait is affected ; in a few after the gait is affected ; and scarcely ever after the patient is unable to walk except by the help of an assistant. The atrophy of the optic nerve in tabes, together with the visual disturbances, is usually much more pronounced and grave than in disseminated sclerosis and nearly always is progressive and leads rapidly to blindness. Dr. Buzzard says that the disc in disseminated sclerosis may acquire a pearly whiteness, but this only exceptionally involves the nasal as well as the temporal side. He has never found it assuming the look of that which is often met with in tabes—" flat, dense and uniform, of a cold, bluish-grey tone, suggesting the idea of its being painted in opaque oil color, with very few retinal vessels visible upon it and a marked absence of minute vascularity."

In general paralysis of the insane there is also a large proportion of cases with optic atrophy. Uhthoff puts it at 50 per cent. and remarks that this is probably too small.

Cases of spastic paraplegia or lateral sclerosis also exhibit a certain proportion of cases affected with optic atrophy, but just what this is has not been determined. It may vary from 5 to 10 per cent.

By this very brief resumé it may be seen that in optic-nerve atrophy alone, to say nothing of those other cases characterized by inflammation of the disc as seen in cerebral tumors, meningitis, cerebral abscess, etc., the neurologist possesses an important accompaniment to the group of symptoms which enter both into the diagnosis and the treatment of a given case. It also suggests the desirability of every neurologist possessing a knowledge of and ability to use the ophthalmoscope.

DISCUSSION.

Dr. L. Howe: Mr. President, I want to venture one word in regard to this subject. We see them in that stage, the chronic stage; we see them in the stage after which it has passed through the acute stage, and I think the importance of that class of cases to which the author has called attention forces itself upon a certain class of practitioners with great intensity. Within the last few weeks the physicians who are attending at the general hospital have had a case which has gone through this acute stage. The girl is practically blind. It is less than a week since I had occasion to see a case with Dr. Hitzel, a child that has gone through the acute stage. It is questionable if it can hear; it can see nothing. I will not stop to consider any question as to the treatment, but I do want to say that a familiarity with the ophthalmoscope, which can be obtained with comparatively a slight degree of effort, would assist one in very many cases, not only as to the question of diagnosis in that acute stage, when it is of importance, but also as to questions of treatment there, which are of importance at that time, and when they are seen afterwards that time has passed—those people are blind. I am very much obliged to the doctor for calling attention to it. I think it is an exceedingly important point.

Dr. F. S. Crego: Mr. President, from the neurological standpoint I want to emphasize two points the doctor has made. The first is, in a large number of cases there is atrophy of the optic nerve early in certain forms of nervous trouble. About two years ago Dr. Hubbell referred a case to me that had been under his observation for five years. The man, five years before, he had noticed had atrophy of the optic nerve. Just then he was beginning to have or did have pronounced locomotor ataxia. The eye changes commenced two or three years before the nervous symptoms appeared. He never had had any of these symptoms of locomotor ataxia, but he had had trouble with his eyes two or three years before. That was a very interesting point to me. I never knew of a case which came on so early and continued so long before the nervous symptoms appear.

The next point I want to emphasize is the one Dr. Howe has spoken of in regard to the ophthalmoscopic examination. Last night a man, whom I had seen before, consulted me, and after talking with him some considerable time I became convinced that he had the initial symptoms of paresis. He didn't have any of the marked symptoms, but he acted very peculiarly and had rather exaggerated ideas of his ability and so on, that gave me the idea that he had paresis. A slight examination of the eye with the ophthalmoscope revealed atrophy of the optic nerve, and I was convinced after that examination that the man eventually would have paresis. And that emphasizes the point Dr. Howe and Dr. Hubbell brought out in the paper. I am exceedingly obliged to Dr. Hubbell for his clear presentation of the matter.

Dr. Hubbell (closing the discussion): You will bear in mind that my paper referred simply to another kind of atrophy—not the acute stage that Dr. Howe has referred to, but without any acute stage—chronic from beginning to end and without any preceding inflammatory symptoms. I am thankful to the doctor for his remarks, inasmuch as he emphasizes the fact that the practitioner ought to use the ophthalmoscope and that the ophthalmoscope can be of the greatest aid to him in diagnosis as well as treatment.

---

Dr. Paul Paquin, of this city, in the *Hot Springs Medical Journal*, says that essence of cinnamon is the first in the list of non-poisonous antiseptics and the strongest, that it is next to bichloride of mercury in its value as an antiseptic, and that the odor of it destroys certain germs.—*St. Louis Clinique*, Dec., 1894.

